# Autism Spectrum Disorder and Narcolepsy: A Possible Connection That Deserves to Be Investigated

**DOI:** 10.3389/fpsyt.2020.00265

**Published:** 2020-04-08

**Authors:** Annio Posar, Paola Visconti, Vincenza Blunda, Fabio Pizza, Giuseppe Plazzi

**Affiliations:** ^1^ IRCCS Istituto delle Scienze Neurologiche di Bologna, UOC Neuropsichiatria Infantile, Bologna, Italy; ^2^ Dipartimento di Scienze Biomediche e Neuromotorie, Università di Bologna, Bologna, Italy; ^3^ IRCCS Istituto delle Scienze Neurologiche di Bologna, UOC Clinica Neurologica, Bologna, Italy

**Keywords:** autism spectrum disorder, narcolepsy, childhood, sleep, psychiatry

## Abstract

Narcolepsy in childhood-adolescence is characterized by a high occurrence of psychiatric comorbidities. The most frequent psychiatric disorders reported in these patients are attention deficit/hyperactivity disorder, depression, anxiety disorder, and schizophrenia. However, narcolepsy can be associated also with introversion, sorrowfulness, feelings of inferiority, impaired affectivity modulation, emotional lability, irritability, aggressiveness, and poor attention, that have been pooled by some authors under a definition of “narcoleptic personality.” Some aspects of this “narcoleptic personality,” and in particular introversion, impaired affectivity modulation, irritability, and poor attention, partially overlap with the clinical features of the individuals with autism spectrum disorder, considering also those that are not regarded as core autism symptoms. Till now, in literature the number of cases affected by both narcolepsy and autism spectrum disorder (seven patients) has been clearly too small to demonstrate the presence of a pathogenetic link between these two conditions, but this possible connection has not yet been adequately investigated, despite the presence of several points in common. The finding of a connection between narcolepsy and autism spectrum disorder could boost the study of possible etiopathogenetic mechanisms shared between these two apparently so distant disorders. Basing on the literature data summarized in this paper, in the diagnostic work-up of a child with narcolepsy it is essential to evaluate also the social-communicative behavior using standardized tools in order to detect the real recurrence of clinical features suggesting an autism spectrum disorder. At the same time, it appears necessary to screen in the individuals with autism spectrum disorder for the possible presence of evoking symptoms of narcolepsy.

## Introduction

Narcolepsy represents a lifelong and disabling neurologic disorder, whose classical main clinical features include: excessive daytime sleepiness; cataplexy, that is the sudden emotion-induced loss of muscle tone; sleep paralysis; hypnagogic (while falling asleep) or hypnopompic (while waking) hallucinations; and disturbed nocturnal sleep (1). Depending on whether or not cataplexy is present, narcolepsy is divided into two types: with (type 1) and without (type 2) cataplexy or cerebrospinal hypocretin-1 deficiency (2). Narcolepsy type 1, unlike type 2, is clearly pathophysiologically related to a severe loss of hypocretin-secreting neurons (located in the hypothalamus), most probably caused by autoimmune mechanisms induced by environmental factors in subjects with a genetic predisposition. The clinical diagnosis of narcolepsy must be supported by instrumental evidence, including findings from nocturnal polysomnography and in particular the multiple sleep latency test, which shows in these patients a decreased mean sleep latency and the appearance of rapid eye movement (REM) sleep within 15 min of falling asleep (3). Brain magnetic resonance imaging basically serves to exclude a secondary form of narcolepsy, due for example to a brain tumor. The prevalence of narcolepsy with cataplexy is around 0.05% in Europe and in North America, but for children epidemiological data are lacking. Age at onset during childhood or adolescence is often reported in a very variable percentage of cases ranging from 5% to about 50% depending on the studies (4).

In narcolepsy a high frequency of psychiatric comorbidities, including neurodevelopmental disorders, has been reported (4, 5). In this paper we addressed the hypothesis of a possible connection between narcolepsy and autism spectrum disorder (ASD), whose main clinical features are early-onset impairments of social communication abilities and the presence of repetitive interests and activities (6). In recent decades, the marked increase in ASD prevalence, which has reached 16.8 per 1,000 children aged 8 years according to a multicenter study in the United States (7), highlights the importance also of possible environmental factors such as pollutants in the etiopathogenesis of this disorder, in addition to indisputable genetic factors (8). The finding of a connection between narcolepsy and ASD, so far not adequately investigated, could boost the study of possible etiopathogenetic mechanisms shared between these two apparently so distant disorders.

## Literature Findings That Could Suggest A Connection Between Narcolepsy And Autism Spectrum Disorder

In both childhood and adulthood, narcolepsy is characterized by a high occurrence of psychiatric comorbidities (4, 5), which, according to Szakács et al., are present in 43% of affected children (9). The most frequent psychiatric disorders reported in these patients include attention deficit/hyperactivity disorder (ADHD), depression, anxiety disorder, and schizophrenia (9, 10). In particular, ADHD, which is the psychiatric disorder most frequently described in association with childhood narcolepsy (11), according to the Diagnostic and Statistical Manual of Mental Disorders (DSM-5, 2013) (6) is part of the large group of neurodevelopmental disorders, characterized by early-onset heterogeneous deficits in personal, social, academic, or occupational functioning. The close link between ADHD and other conditions included within the neurodevelopmental disorders is suggested also by the frequent comorbidity between these conditions. For example, ADHD is present also in 33–37% of cases with ASD (12). Moreover, it should be noted that also other conditions belonging to the neurodevelopmental disorders according to the DSM-5 classification, that is specific learning disorders (characterized by an impairment in reading, written expression, and/or mathematics) (6), recur with high frequency in children with narcolepsy, probably due to subtle and heterogeneous cognitive impairments, in the absence of intellectual disability (13).

Narcolepsy can be associated also with less conspicuous problems, including introversion, sorrowfulness, feelings of inferiority, impaired affectivity modulation, emotional lability, irritability, aggressiveness, and poor attention that some authors have defined as the “narcoleptic personality” (4). This type of symptomatology is very important to know and to take into consideration, as it can lead to diagnostic errors suggesting the presence of a primary psychiatric disorder and consequently favoring a diagnostic delay that has been estimated to be around 14 years both in Europe and in the United States (14–16). In this regard, it is no coincidence that in the past, for decades, various authors have supported the hypothesis of a psychogenic etiology of narcolepsy (5). Apart from the issue of possible misdiagnosing, the presence of a psychiatric symptomatology can in itself significantly impair the quality of life of the affected individuals, regardless of the aforementioned core symptoms of narcolepsy. Note that some aspects of the so-called narcoleptic personality, and in particular introversion, impaired affectivity modulation, irritability, and poor attention, partially overlap with the clinical features of the individuals with ASD, considering also those that are not regarded as core autism symptoms.

As further confirmation of the psychosocial impairment in early-onset narcolepsy, we mention the work of Rocca et al., who studied behavior and quality of life in 29 children and adolescents with narcolepsy type 1 compared with 39 matched subjects affected by idiopathic epilepsy and 39 matched healthy controls using self-administered questionnaires (Child Behavior Checklist, and Pediatric Quality of Life Inventory). The authors found a higher recurrence of anxiety/depression, withdrawn attitude, social, thinking, and attention problems, somatic complaints, and aggressive behaviors in the patients with narcolepsy type 1 compared to controls, suggesting a relationship with sleepiness. Parents of the patients also reported worse psychosocial health (17).

It has been hypothesized that the psychosocial impairment in childhood narcolepsy could not only be due to the excessive sleepiness, but also to other factors such as the presence of hypnagogic or hypnopompic hallucinations, which can cause significant distress in those who experience them (18).

It is possible that at the origin of the association between narcolepsy and psychiatric disorders there is a sharing of pathogenetic mechanisms: see in particular the dysfunction of the hypocretinergic system, called into question, as mentioned above, in the pathogenesis of narcolepsy type 1, but hypothetically involved also in the pathogenesis of several psychiatric disorders including anxiety, depression, and schizophrenia (19). Also, beyond pathological conditions, there are some data to support the hypothesis that the hypocretinergic system plays a role in modulating social behavior in humans (20).

As to schizophrenia, in the past ASD and childhood onset schizophrenia have long been considered as clearly separated or even mutually exclusive conditions: in fact, within the diagnostic criteria of the DSM-III (1980) for infantile autism there is also the “absence of delusions, hallucinations, loosening of associations, and incoherence as in schizophrenia” (21). But more recently, ASD and schizophrenia have been reported to share multiple phenotypic findings and risk factors (genetic and environmental), as well as to co-occur at high rates (22). Not by chance, a recent review has shown significantly higher autistic symptoms in the subjects with schizophrenia than in the healthy controls (23). Therefore, the existence of a link between narcolepsy and schizophrenia on one side, and between ASD and schizophrenia on the other side, indirectly suggests the presence of a possible link also between narcolepsy and ASD.

Considering the high prevalence of sleep problems (and in particular of insomnia) in the individuals with ASD as well as the known role of hypocretin in the wake-sleep circadian rhythm, Messina et al. hypothesized that patients with ASD may have a hypocretinergic system dysfunction consisting of an increased activity (24), probably caused by an amygdala dysfunction, the latter suggested by functional magnetic resonance imaging studies in adults and children with ASD (25–27). In this perspective, it could be interesting to study the hypothesis that at least in a subgroup of subjects with ASD during the life there may be an initial excess of activity of the hypocretinergic system (to which the problems of insomnia are fundamentally correlated), followed by a normalization of this system or even, more rarely, by a sort of exhaustion leading to a deficit/absence of hypocretin with the possible appearance of narcoleptic symptoms. And then, perhaps, it is not a coincidence that usually in childhood the age at onset of ASD tends to be earlier than that of narcolepsy. As can be easily imagined, there are no longitudinal studies that investigate the levels of hypocretin in the cerebrospinal fluid of ASD patients over their life course. In addition, it should be noted that an amygdala dysfunction has also been shown in adults and children with narcolepsy type 1 through functional magnetic resonance imaging studies and this finding represents a further point of contact with ASD (28–32). On the other hand, also widespread white matter connectivity abnormalities, reported both in ASD and in narcolepsy type 1 patients using structural magnetic resonance imaging (33, 34), could represent further common pathogenetic mechanisms suggesting a connection between these two disorders.

## Co-Occurrence Of Narcolepsy And Autism Spectrum Disorder

From the above considerations, the question arises whether there could be a link between narcolepsy and ASD. A first approach to answer this question is to see if these two nosographic entities can coexist in the same person. So far, the concomitant presence of these two conditions in the same individual has been explicitly described overall only in seven cases with childhood narcolepsy: in one patient with pervasive developmental disorder—not otherwise specified reported briefly by Szakacs et al. (9) and in six cases with Asperger syndrome, of which two mentioned by Huang et al. (10) and, more recently, four described by Prihodova et al. (35).

The case mentioned by Szakacs et al. is included in a series of 38 children and adolescents with narcolepsy evaluated with the following tools for the psychological and psychiatric assessment: psychometric tests (Wechsler Scales), semistructured interviews (Development and Well-Being Assessment; Positive and Negative Syndrome Scale; Attention Deficit Hyperactivity Disorder Rating Scale), and the Autism Spectrum Screening Questionnaire. Unfortunately, the Autism Diagnostic Observation Schedule, which today is considered the gold standard tool for the diagnosis of ASD, has not been mentioned (9). According to the Diagnostic and Statistical Manual of Mental Disorders, 4th ed., Text Revision (DSM-IV-TR) (2000), pervasive developmental disorder–not otherwise specified represents, among the pervasive developmental disorders, a sort of residual category (i.e., atypical autism) that includes those cases who do not meet all the criteria for the autistic disorder (i.e., classic autism) due to late onset and/or atypical/subthreshold symptomatology (36). However, the description of Szakacs's patient with pervasive developmental disorder–not otherwise specified appears very incomplete, so much so that, for example, the intellectual level of this case is not known. Further, other two patients reported by Szakacs et al. were positive on the Autism Spectrum Screening Questionnaire screening, but they did not receive a definite diagnosis in the area of ASDs (9).

Huang et al. mentioned that out of 10 adolescents affected by narcolepsy with cataplexy and associated schizophrenia, two had as comorbidity an Asperger syndrome, without giving further details. The authors stated that comorbidities were determined using the criteria of the DSM-IV-TR: it follows that their intellectual functioning was not impaired, since cognitive development is not significantly delayed in Asperger syndrome according to the DSM-IV-TR criteria (10).

Prihodova et al. described with more details four children with narcolepsy type 1 who have been diagnosed also with Asperger syndrome according to the psychological and psychiatric assessment, including the Autism Diagnostic Observation Schedule (module 3, utilized for verbally fluent individuals), Developmental NEuroPSYchological Assessment, Second Edition (part: Affect Recognition), and Wechsler Intelligence Scale for Children—Third Edition (35). Asperger disorder is another diagnostic category included in the DSM-IV-TR within the pervasive developmental disorders, characterized by qualitative abnormalities in social interaction and by restricted and stereotyped interests and activities, but at the same time without language delay or intellectual disability (36). All four patients reported by Prihodova et al. showed an early onset impairment of social interactions and had an average or above-average intelligence quotient, with significantly uneven profiles in the Wechsler scale subtests. Note that in two of these four cases also ADHD was associated: in this regard, see the aforementioned high frequency of co-occurrence among the neurodevelopmental disorders. According to Prihodova et al., the symptoms of narcolepsy type 1 and the psychosocial functioning impairment could exacerbate or uncover the autistic symptomatology. Therefore, the authors highlighted the importance of a psychological and psychiatric assessment including an evaluation of social abilities in children with narcolepsy (35).

It should be stressed here that the DSM-5 (2013) has eliminated the five diagnostic subcategories of pervasive developmental disorders proposed by the DSM-IV-TR (including precisely both pervasive developmental disorder–not otherwise specified and Asperger disorder) (36), considering only the all-embracing category of the ASD (6). Therefore, the seven mentioned patients reported by Szakacs et al. (9), Huang et al. (10), and Prihodova et al. (35) nowadays should be presumably diagnosed with ASD according to the criteria of the most recent edition of the DSM (6). Note that this innovation of the DSM-5 has not obtained unanimous consensus among the authors, since it entails the risk of a less detailed clinical characterization of the cases affected by autism (37).

Recently, Quaedackers et al. (38) studied social functioning in a sample of 53 children with narcolepsy type 1 compared to 64 matched healthy children using the Social Responsiveness Scale and the Child Behavior Checklist given to parents. Compared to controls, patients scored significantly higher (and therefore more pathological) on the Social Responsiveness Scale total score as well as on the sum of the Child Behavior Checklist subscales related to social functioning. Out of 53 patients, 24 (45.3%) reported at least mild-to-moderate problems in social functioning compared to only seven controls (10.9%) (p < 0.001). A Social Responsiveness Scale T score above 75, which is strongly associated with a diagnosis of ASD, prevailed significantly in patients (11: 20.8%) than in controls (1: 1.6%). Among the patients, on the Social Responsiveness Scale females reported mild-to-moderate problems in social functioning significantly more often compared to males (36). Although the cases reported by these authors have not been subjected to the gold standard tests (e.g.: Autism Diagnostic Observation Schedule) for the diagnosis of ASD, but only to a screening test as is the Social Responsiveness Scale that can give a positive result also in disorders other than ASD such as ADHD or anorexia nervosa, this paper further emphasizes the possibility of the coexistence, and therefore of a link, between narcolepsy and ASD. It should be noted that the intellectual functioning of patients with narcolepsy was not studied in this paper, not even in those with a Social Responsiveness Scale T score above 75 (38).

## Conclusions

Till now, in literature the number of cases affected by both childhood narcolepsy and ASD has been clearly too small to demonstrate the presence of a pathogenetic link between these two conditions, but this possible connection has not yet been adequately investigated, despite the presence of several points in common between narcolepsy and ASD. For this purpose, in the diagnostic work-up of a child with narcolepsy it is essential to evaluate also the social-communicative behavior using standardized tools in order to detect the real recurrence of clinical features suggesting ASD. At the same time, it appears necessary to screen in the individuals with ASD for the possible presence of evoking symptoms of narcolepsy and, if present, to study them with suitable diagnostic tools (including multiple sleep latency test). A systematic work of this kind could provide epidemiologically reliable findings on the possible connection between narcolepsy and ASD.

Further, a deeper investigation on shared clinical features, time course, and treatment effects of both these disorders could shed light on common pathogenetic mechanisms.

Finally, studying in depth the presence of a family history for neurodevelopmental disorders, including ASD, in the individuals with narcolepsy could provide data in favor of the existence of a heterogeneous spectrum of conditions, possibly sharing a common cerebral dysfunction, as suggested already in 1998 by Cohen who reported an ASD child with a familiarity for narcolepsy and multiple sclerosis (39). In this regard, even the hypothesis of a common immune dysregulation could be a further mechanism connecting narcolepsy and ASD, since also in the latter there are various data suggesting a dysfunction of the immune system (40).

**Tables 1** and **2** summarize respectively the state of the art and the agenda of future research lines about the possible connection between narcolepsy and ASD.

**Table 1:** Possible connection between narcolepsy and autism spectrum disorder: the state of the art.

- High occurrence of psychiatric comorbidities in childhood narcolepsy (including neurodevelopmental disorders), particularly attention deficit/hyperactivity disorder, depression, anxiety disorder, and schizophrenia. - Narcolepsy can be associated also with psychiatric symptoms (“narcoleptic personality”) of which some aspects (particularly introversion, impaired affectivity modulation, irritability, and poor attention) partially overlap with the clinical features of autism spectrum disorder, considering also those that are not regarded as core autism symptoms. - Co-occurrence of narcolepsy and autism spectrum disorder in the same individual reported in seven cases so far. - Possible connection between narcolepsy and autism spectrum disorder not yet adequately investigated despite several common features.

**Table 2:** Possible connection between narcolepsy and autism spectrum disorder: the agenda of future research lines.

- In children with narcolepsy: evaluation of the social-communicative behavior using standardized tools to detect the co-occurrence of an autism spectrum disorder. - In individuals with autism spectrum disorder: screening for the presence of evoking symptoms of narcolepsy and, if present, studying them with suitable diagnostic tools. - Performing a deeper investigation on shared clinical features, time course, and treatment effects of narcolepsy and autism spectrum disorder. - Studying in depth family history for neurodevelopmental disorders (including autism spectrum disorder) in the individuals with narcolepsy.

## Author Contributions

AP conceived the article, carried out the literature review, and drafted the manuscript. PV, VB, FP, and GP contributed to the literature review and critically revised the manuscript.

## Conflict of Interest

The authors declare that the research was conducted in the absence of any commercial or financial relationships that could be construed as a potential conflict of interest.
